# The one-week prevalence of neck pain and low back pain in post-secondary students at two Canadian institutions

**DOI:** 10.1186/s12998-023-00496-y

**Published:** 2023-07-31

**Authors:** Alexandra Campbell, Dan Wang, Krystle Martin, Pierre Côté

**Affiliations:** 1 Faculty of Health Sciences, Ontario Tech University, Oshawa, Canada; 2 Institute for Disability and Rehabilitation Research, Ontario Tech University, Oshawa, Canada

**Keywords:** University students, Cross-sectional study, Neck pain, Low back pain

## Abstract

**Background:**

Low back and neck pain are common in the general population, but the prevalence among Canadian post-secondary students is not well known. We aimed to determine the one-week prevalence of neck pain (NP) and low back pain (LBP) among postsecondary students in Canada.

**Methods:**

We conducted a cross-sectional study of students enrolled in the Faculty of Health Sciences and Faculty of Education at Ontario Tech University, and the Canadian Memorial Chiropractic College (CMCC) in the Fall of 2017. Neck and low back pain intensity in the past week were measured with the 11-point numerical rating scale. We report the cumulative, gender- and institution-specific one-week prevalence (95% CI) of any pain (1–10/10) and moderate to severe pain (≥ 3/10).

**Results:**

The one-week prevalence of any neck pain ranged from 45.4% (95% CI: 38.4, 52.4) in the Faculty of Education to 76.9% (95% CI: 72.9, 80.4) at CMCC. The one-week prevalence of neck pain ≥3/10 ranged from 44.4% (95% CI: 37.5, 51.4) in the Faculty of Education to 58.4% (95% CI: 54.0, 62.7) at CMCC. The one-week prevalence of any low back pain ranged from 60.9% (95% CI: 53.8, 67.5) in the Faculty of Education to 69.0% (95% CI: 64.8, 73.0) at CMCC, and the one-week prevalence of low back pain ≥ 3/10 ranged from 47.8% (95% CI: 43.4, 52.2) at CMCC to 55.1% (95% CI: 51.2, 58.9) in the Faculty of Health Sciences. The prevalence of any back or neck pain and pain ≥ 3/10 was consistently higher in females than males, with the largest difference seen for neck pain at CMCC.

**Conclusion:**

Most post-secondary students in our samples experienced LBP and NP in the past week. Overall, the one-week prevalence of NP and LBP was higher among chiropractic students and among females. This study should draw attention to school administrators about the burden of NP and LBP in post-secondary students.

**Supplementary Information:**

The online version contains supplementary material available at 10.1186/s12998-023-00496-y.

## Background

Neck pain (NP) and low back pain (LBP) are two of the most common musculoskeletal disorders in the general population [1]. Globally, the age-standardized point prevalence of NP is 2696.5 per 100,000 [2] and the global age-standardized point prevalence of LBP is 6972.5 per 100,000 [3]. NP and LBP are chronic and recurrent conditions associated with disability in adolescents and young adults [1, 4, 5]. In fact, according to the 2019 Global Burden of Disease study, LBP ranks 7th in people aged 10–24 years and fourth for those aged 25–49 years [6].

The current evidence suggests that the prevalence of NP and LBP may be particularly high in post-secondary students [1, 7–10]. In Canada, post-secondary education refers to education completed beyond the secondary (high school) level. Post-secondary education institutions include colleges and universities [11]. Most students enrolled in postsecondary institutions in Canada are between the ages of 20–24 years [12]. A study conducted in medical students in Saudi Arabia reports that the one-week prevalence was 44% for NP and 33% for LBP [13]. Furthermore, a study of health science students in Saudi Arabia reported a 12-month prevalence of 48% for LBP [10]. In Malaysian medical students, the one-week prevalence was 24% and 27% for NP and LBP, respectively [9]. A meta-analysis of studies conducted with nursing and medical students reported a 12-month prevalence of LBP of 44% (95% CI: 27%, 61%) in medical students and 55% (95% CI: 44%, 62%) in nursing students [14].

Factors associated with NP or LBP include sleep quality and physical inactivity. While the association between these variables was not investigated in this paper, the presence and severity of these variables were described. Previous research suggests that sleep problems may be associated with back pain [15]. A systematic review assessing psychosocial variables and NP in adolescents reported limited evidence suggesting that insufficient quality and quantity of sleep was associated with NP in adolescents [16]. The literature is inconclusive regarding physical activity and sedentary behaviour on NP or LBP [9, 10, 17–19]. Describing these characteristics in our study is important to contextualize our study.

To our knowledge, the prevalence of NP and LBP in Canadian post-secondary students remains unknown. Understanding the prevalence of NP and LBP in this population is important because young adulthood is a critical time when the onset or experience of musculoskeletal pain could be prevented [1, 20]. Moreover, it is a period when education about NP and LBP could potentially reduce future disability related to spinal pain [5, 21]. However, the prevalence of NP and LBP in Canadian post-secondary students remains unknown. Therefore, we aimed to describe the cumulative and gender-specific one-week period prevalence of NP and LBP in students enrolled in two post-secondary institutions in Canada.

## Methods

The reporting of our cross-sectional study complies with the Strengthening the Reporting of Observational Studies in Epidemiology (STROBE) Statement (Appendix 1) [22].

### Design

We designed and conducted the Mental Health and Wellness Study, a cross-sectional study of post-secondary students enrolled at Ontario Tech University and at the Canadian Memorial Chiropractic College (CMCC) in the fall of 2017. Students were eligible if they were enrolled full-time in the Faculty of Health Sciences or the Faculty of Education at Ontario Tech University or the CMCC and were 18 years of age or older [23–25].

### Context

Ontario Tech University is primarily an undergraduate university located in Oshawa, Ontario, Canada. Ontario Tech University offers a range of programs in six faculties: Faculty of Science, Faculty of Engineering, Faculty of Social Science and Humanities, Faculty of Health Sciences, Faculty of Business and Information Technology and the Faculty of Education. Ontario Tech University has a population of domestic, international, and college transfer students. In 2017, the Faculty of Health Sciences included 1931 individuals enrolled in undergraduate degrees (Bachelor of Health Science [Honours], and Bachelor of Allied Health Science [Honours]) in a range of programs, including Public Health, Kinesiology, Medical Laboratory Science, Nursing, and Allied Health Science. During the same period, the Faculty of Education included 268 students pursuing a Bachelor of Education degree.

CMCC is Canada’s only English-speaking chiropractic academic institution. It is located in Toronto, Ontario, Canada and offers professional education for students pursuing a Doctor of Chiropractic degree. In 2017, CMCC had a total enrollment of 766 students. Students enrolled at CMCC previously completed at least three years of undergraduate university education. The CMCC education program includes four years of courses and clinical training.

### Recruitment

Students from Ontario Tech University were recruited during three consecutive waves, from mid-September to October of 2017. Students, enrolled in 27 mandatory classes from the Faculty of Health Sciences and the Faculty of Education were invited to participate in the first wave of recruitment. Compulsory courses were selected to ensure that the largest number of students were provided with the opportunity to participate and had the chance to complete the survey during class time. Recruitment was standardized and included three steps. First, the professor or instructor for each class read a script to introduce the research team. Second, the professor or instructor left the room. The research team addressed the class and delivered a five-minute presentation that outlined the purpose of the study, explained the informed consent process, and offered information for community and school-based mental health services if needed. Finally, the students were encouraged to ask questions and then given 15 minutes to complete the electronic questionnaire in class. The research assistant administered the questionnaire and provided support for any students if they had difficulty interpreting the question. The research assistant had no relationship to the students. The informed consent process emphasized that participation was voluntary and would not have any impact on their grades or course (especially with the instructor leaving the room).

For the second wave of recruitment, the professor or instructor from each mandatory class sent a follow-up email to all students inviting them to enrol and complete the study questionnaire by clicking on a link. Similarly, the final wave of recruitment included an email reminder from the Dean, who invited all students to enrol and complete the online questionnaire.

Students from CMCC were recruited during mandatory classes for those enrolled in their program’s first, second, and third years. Students enrolled in the fourth year of education were recruited during clinic rounds. The in-person recruitment was supplemented with online announcements (i.e., Facebook posts), a post in CMCC’s newsletter, and posters within the CMCC facilities. Recruitment began at the end of October 2017, and online enrollment was possible until mid-November 2017.

### Data collection

The online self-administered questionnaire included valid and reliable measurement tools, including the Physical Activity and Sedentary Behaviour Questionnaire (PASB-Q) for physical activity and sedentary behaviour [26], The Household Food Security Survey Model (HFSSM) for food security [27], The Pittsburgh Sleep Quality Index (PSQI) for sleep quality [28], medical diagnosis, and sociodemographic factors [23] (Appendix 2). We also used questions from Statistics Canada and the Canadian Community Health Survey to measure medically diagnosed conditions [29]. The collected data was stored securely on Google Forms and Google Drive at Ontario Tech University. A similar protocol was followed at CMCC.

### NP and LBP measurement

The questionnaire included a body diagram identifying the neck and low back region. Students reported whether they had experienced NP and LBP in the past week. Those who reported pain were asked to rate the intensity of their NP and LBP on an 11-point Numeric Rating Scale (NRS), where 0/10 indicated no pain and 10/10 indicated the worst pain possible [30]. The NRS is a valid and reliable instrument to measure pain intensity in the adult population [31–37]. The evidence suggests that the NRS has adequate test-retest reliability (intraclass correlation coefficient 0.58 to 0.93) [35, 36].

### Statistical analysis

We described the sociodemographic, lifestyle and health-related characteristics of our samples. We computed the cumulative and gender-specific one-week prevalence and 95% confidence intervals (CI) of: (1) any (1–10/10 on the NRS) NP and LBP; and, (2) moderate to severe NP and LBP (≥3/10 on the NRS) for students in each institution [38]. We selected a cut-point ≥3/10 because it is recommended to differentiate participants with and without clinically meaningful levels of pain [38, 39]. For the prevalence of any NP or LBP, the numerator included those who responded “yes” to experiencing any NP or LBP in the preceding week, and the denominator was the total sample size. Similarly, for the computation of moderate to severe pain prevalence, the numerator included those who rated their pain ≥3/10, and the denominator included the total sample. The epiR package (version 2.0.41) in R was used to conduct the analysis [40].

We assessed the presence of participation bias by comparing the age, gender, and year of study of the students in our sample to all students enrolled in each faculty/institution.

## Results

### Participation

At Ontario Tech University, participation in the Faculty of Education was 77% (207/268), and 34% (675/1931) in the Faculty of Health Sciences. Of the 766 eligible students at CMCC, 510 completed the survey, with a participation rate of 67%. The source population comprises of all students enrolled in the Faculty of Health Science, the Faculty of Education, and CMCC at the time of data collection. A comparison of our samples and the source populations suggest that (1) the sample from the Faculty of Education was younger than the population (mean ± SD: 25.6 ± 4.8 versus 28.0), (2) more females participants were recruited at CMCC (60.0% versus 55.4%), and at the Faculty of Health Science (79.9% versus 75.9%), and, (3) first-year students were overrepresented in the Faculty of Health Sciences at Ontario Tech University (see Table 1).

**Table 1:** A comparison of the study samples with the sourced population

	Ontario Tech University				Canadian Memorial Chiropractic College	
	Faculty of Health Sciences		Faculty of Education			
Variable	Population (n = 1931)	Sample (n = 675)	Population (n = 268)	Sample (n = 207)	Population (n = 766)	Sample (n = 510)
Age (SD)	24	22.1 (5.5)	28	25.6 (4.8)	25	24.56 (2.7)
Gender = Female	75.9%	79.9%	66.7%	68.1%	55.4%	60.0%
Year of Study						
1	18.1%	25.3%	52.4%	33.3%	26.0%	25.7%
2	26.3%	25.6%	47.6%	34.8%	24.8%	26.9%
3	27.2%	21.0%	0%	0%	24.3%	29.2%
4	25.5%	26.5%	0%	0%	24.9%	18.2%
5+	2.9%	1.5%	0%	31.9%^1^	0%	0%

^1^ – Students may have been confused by the question and responded as year 5 + if they had completed their undergraduate degree at Ontario Tech and were continuing with their Bachelor of Education at the same institution, thereby making them assume they are year 5 + total, compared to year 1 or 2 of the second program

### Sample characteristics

Most participants in the Faculty of Health Sciences were female (79.9%) (Table 1). The average age of participants at Ontario Tech University ranged from 22.1 to 25.6 years, with the Faculty of Education presenting as the higher age. More than half (52.6%) of the Faculty of Health Sciences sample reported having been diagnosed with a medical condition by a healthcare provider (Table 2), with allergies being the most common (29.0%). In the Faculty of Education, 68.1% of participants identified as female (Table 1). Regarding medical conditions, 54.1% in the Faculty of Education reported having been diagnosed with a medical condition by a healthcare provider, and, similar to the Faculty of Health Sciences, allergies were the most common (29.0%). Both of the samples at Ontario Tech University engaged in sedentary behaviour (> 7 h), with 100% of the students meeting this threshold. Furthermore, sitting time (> 3 h) was lower at Ontario Tech University, ranging from 35.7 to 38.1%. Moderate to vigorous aerobic activity (2 + days a week) at Ontario Tech University was slightly higher than CMCC, ranging from 2.9% to 5.8%. Furthermore, most students in the Faculty of Health Science (65.8%) and the Faculty of Education (61.4%) reported poor sleep quality.

**Table 2:** Sample Characteristics

	Ontario Tech University		Canadian Memorial Chiropractic College (n = 514)
	Faculty of Health Science (n = 675)	Faculty of Education (n = 207)	
**Medical Condition**	355 (52.6%)	112 (54.1%)	238 (46.7%)
Allergy	196 (29.0%)	60 (29.0%)	128 (25.1%)
Arthritis	9 (1.3%)	< 5 (1.0%)	8 (1.6%)
Asthma	98 (14.5%)	22 (10.6%)	62 (12.2%)
ADD-H-D^1^	21 (3.1%)	11 (5.3%)	13 (2.5%)
IBS^2^	23 (3.4%)	8 (3.9%)	21 (4.1%)
CFS^3^	0	0	< 5 (0.6%)
Eating Disorder	19 (2.8%)	< 5 (0.5%)	7 (1.4%)
High Blood Pressure	< 5 (0.6%)	5 (2.4%)	< 5 (0.4%)
Ulcers	6 (0.9%)	0	< 5 (0.4%)
Migraine	61 (9.0%)	12 (5.8%)	38 (7.5%)
Mood Disorder	66 (9.8%)	21 (10.1%)	30 (5.9%)
Scoliosis	19 (2.8%)	6 (2.9%)	20 (3.9%)
STI^4^	< 5 (0.4%)	5 (2.4%)	11 (2.5%)
Other	22 (3.3%)	7 (3.4%)	8 (1.6%)
**Sedentary Time > 7 h**	675 (100%)	207 (100%)	514 (100%)
**Sitting Time > 3 h**	257 (38.1%)	74 (35.7%)	253 (49.6%)
**Moderate-Vigorous Aerobic** Meet Guidelines (2 + days/week)	39 (5.8%)	6 (2.9%)	8 (1.6%)
**Muscle Strengthening** Meet Guidelines (2 + days/week)	257 (38.1%)	57 (27.7%)	312 (61.5%)
**Food Insecure/Hungry** The Household Food Security Survey Model (≥ 2 affirmatives)	58 (7.8%)	15 (7.2%)	28 (0.05%)
**Poor Sleep** The Pittsburgh Sleep Quality Index (≥ 5/21)	444 (65.8%)	127 (61.4%)	275 (53.9%)

1 – Attention Deficit Hyperactivity Disorder, 2 – Irritable Bowel Syndrome, 3 – Chronic Fatigue Syndrome, 4 – Sexually Transmitted Diseases

Most participants at CMCC were female (60.0%) but accounted for fewer female participants in comparison to Ontario Tech University (79.9% and 68.1%) (Table 1). The CMCC sample had a younger average age than the Faculty of Education sample but included the highest percentage of male participants. The average age of the CMCC sample was 24.6 (SD: 2.7). Less than half of the sample at CMCC reported being diagnosed with a medical condition (46.7%), and allergies were the most common condition (25.1%). The entire CMCC sample also engaged in sedentary behaviours (100%) and had the largest proportion of participants that engaged in sitting time > 3 hours. CMCC students had the lowest levels of aerobic activity (2 + days a week of moderate to vigorous aerobic exercise) at 1.6%. The CMCC sample reported the highest percentage of students meeting the strengthening guidelines (2 + days a week) at 61.5%. Compared to the Ontario Tech University sample, the proportion of CMCC students who reported poor sleep quality was lower (53.9%).

### One-week prevalence of NP

The one-week prevalence of any NP ranged from 45.4% (95% CI: 38.4, 52.4) in the Faculty of Health Sciences to 76.9% (95% CI: 72.9, 80.4) at CMCC (Table 3). The prevalence of moderate to severe NP (≥3/10) ranged from 44.4% (95% CI: 37.5, 51.4) in the Faculty of Education to 58.4% (95% CI: 54.0, 62.7) at CMCC. Females, compared to males, had a higher prevalence of any NP, ranging from 68.1% (95% CI: 63.9, 72.0) to 85.3% (95% CI: 80.8, 89.0) (Table 3), while it ranged from 45.3% (95% CI: 36.4, 54.3) to 64.5% (95% CI: 57.5, 71.1) in males. Female students also reported a higher prevalence of moderate to severe NP (≥ 3/10), with the highest difference in the CMCC sample with a one-week prevalence of 70.6% (95% CI: 65.1, 75.6), compared to the prevalence among Ontario Tech University students which ranged from 47.5% (95% CI: 39.0, 56.0) to 57.1% (95% CI: 52.8, 61.3). The prevalence for moderate to severe NP (≥ 3/10) in males ranged from 32.8% (95% CI: 24.7, 41.6) to 40.4% (95% CI: 33.5, 47.4).

**Table 3:** The one-week overall and gender-specific prevalence (95% confidence interval) of neck pain and low back pain in three samples of Canadian post-secondary students

	Ontario Tech University		Canadian Memorial Chiropractic College
	Faculty of Health Sciences	Faculty of Education	
**Any Neck Pain**			
All	63.7% (59.9, 67.3)	45.4% (38.4, 52.4)	76.9% (72.9, 80.4).
Female	68.1% (63.9, 72.0)	56.0% (47.4, 64.3)	85.3% (80.8, 89.0)
Male	45.3% (36.4, 54.3)	50.7% (38.0, 63.3)	64.5% (57.5, 71.1)
**Neck Pain ≥ 3/10**			
All	52.1% (48.3, 55.9)	44.4% (37.5, 51.4)	58.4% (54.0, 62.7)
Female	57.1% (52.8, 61.3)	47.5% (39.0, 56.0)	70.6% (65.1, 75.6)
Male	32.8% (24.7, 41.6)	36.9% (25.2, 49.8)	40.4% (33.5, 47.4)
**Any Low Back Pain**			
All	67.4% (63.7, 70.9)	60.9% (53.8, 67.5)	69% (64.8, 73.0)
Female	70.5% (66.4, 74.3)	63.1% (54.5, 71.0)	72.5% (67.1, 77.4)
Male	53.9% (44.8, 62.7)	55.3% (42.5, 67.7)	64% (57.0, 70.6)
**Low Back Pain ≥ 3/10**			
All	55.1% (51.2, 58.9)	51.2% (44.1, 58.1)	47.8% (43.4, 52.2)
Female	58.6% (54.3, 62.8)	53.9% (45.3, 62.3)	51.6% (45.8, 57.3)
Male	42.1% (33.5, 51.2)	44.6% (32.2, 57.4)	42.3% (35.4, 49.4)

### One-week prevalence of LBP

The one-week prevalence of any LBP ranged from 60.9% (95% CI: 53.8, 67.5) in the Faculty of Education to 69% (95% CI: 64.8, 73.0) at CMCC (Table 3). Regarding low back pain rated ≥ 3/10, the one-week prevalence ranged from 47.8% (95% CI: 43.4, 52.2) at CMCC to 55.1% (95% CI: 51.2, 58.9) in the Faculty of Health Sciences. Female students had a higher prevalence of any LBP ranging from 63.1%(95% CI: 54.5, 71.0) to 72.5% (95% CI: 67.1, 77.4) at CMCC (Table 3). In males, the one-week prevalence of any LBP ranged from 53.9% (95% CI: 44.8, 62.7) at the the Faculty of Health Sciences to 64% (95% CI: 57.0, 70.6) at CMCC. Females also had a higher prevalence of moderate to severe (≥ 3/10) LBP ranging from 51.6% (95% CI: 45.8, 57.3) at CMCC to 58.6%% (95% CI: 54.3, 62.8) at the the Faculty of Health Sciences. In contrast, males had a one-week prevalence of moderate to severe (≥ 3/10) LBP ranging from 42.1% (95% CI: 33.5, 51.2) at Faculty of Health Sciences to 44.6% (95% CI: 32.2, 57.4) in the Faculty of Education.

## Discussion

The results from our study show that the one-week prevalence of LBP and NP is high among students enrolled in two Canadian post-secondary institutions. Excluding mild pain, the prevalence remains high in all samples. This could be possibly explained by potential risk factors present in this age group, like daily computer use [1, 9].

Compared to previous studies from Saudi Arabia and Malaysia [9, 10, 13, 41], the prevalence in our study samples was higher, despite having populations similar in age and occupation (student). However, our results are consistent with the Global Burden of Disease study results, indicating that the prevalence of LBP and NP was highest in North America than in other countries globally [3, 42]. Additionally, the one-week prevalence of any NP and LBP and pain rated ≥ 3/10 was higher among females than males across all three samples. These results align with previous work that found a higher prevalence of musculoskeletal pain in women compared to men [43–45]. One reason for this difference may be due to pain perception. There is evidence that sex differences in pain perception can be attributed to oestrogen, a hormone that regulates the menstrual cycle and could exacerbate pain [46]. Another potential reason for this finding could be due to the higher participation of females than males across the three samples (79.9% in the Faculty of Health Sciences, 68.1% in the Faculty of Education, and 60% at CMCC). This does align with work demonstrating that survey respondents are more likely to be female [47]. The higher proportion of females could be leading to the higher prevalence of NP, and LBP demonstrated in our sample.

Across study samples, the one-week prevalence for LBP and NP was higher at CMCC than at Ontario Tech University. Within Ontario Tech University, the prevalence of any NP and LBP, as well as pain ≥ 3/10, was higher in the Faculty of Health Sciences than in the Faculty of Education. Previous literature suggests that the dose of physical activities is associated with back pain among students, with regular physical activity reducing musculoskeletal pain [48]. Interestingly, a previous study found that CMCC students are physically active, with 72% reporting that they met physical activity guidelines [49], thereby potentially negating a hypothesis that a potential lack of activity could be contributing to their prevalence of LBP and NP. But, some evidence suggests that while physical activity in leisure time may reduce LBP, occupational activity, such as chiropractic, could be strenuous enough to increase their risk of chronic LBP [50]. Although this physical load may differ from working full-time as a chiropractor, students at CMCC may be engaging in more strenuous physical activity through their education than the Ontario Tech University sample (Table 2). For example, students at CMCC engage in clinical training starting in year one and amass a total of 1,775 hours of training by the end of their fourth year [51]. Furthermore, being in a health-related discipline may affect the health self-awareness of students which may be associated with higher reporting of NP and LBP. Additionally, the CMCC sample did differ from Ontario Tech University in sitting time (> 3 hours) and whether they engaged in moderate to vigorous aerobic exercise more than 2 days a week (Table 2).

Young adult students may differ from young non-students adult across a range of lifestyle factors (e.g., stress, sleep, and sedentary behaviour), and these differences may be associated with the prevalence of LBP and NP. For example, the 2020 Canadian Community Health Survey suggests that almost 80% of adults aged 18–34 met the sleep guidelines (between 7 and 9:59 h), while only 59% of post-secondary students met the sleep guidelines [52, 53]. Poor sleep quality is a possible risk factor for NP and LBP in this population [54]. As evidenced in Table 2, most students had poor-quality sleep (ranging from 53.9 to 65.8%). Furthermore, students may have higher perceived stress than their non-student peers [55], and study-related stress may be associated with LBP [56]. Finally, sedentary behaviour in this population may have contributed to their experience of LBP or NP. Previous work found that undergraduate students who engage in sedentary behaviour for at least 12 hours per day may differ from non-students who have more flexible schedules [57]. This is important because those reporting more sedentary hours may be more likely to report musculoskeletal pain [58]. It is important to note that the entire sample (100%) did engage in sedentary behaviour (> 7 h), most participants (94.2%–98.4%) did not meet the guidelines of 2 + days a week of moderate to vigorous aerobic activity (Table 2). This suggest that sleep, sedentary behaviour, and physical inactivity may impact the report of NP and LBP in our population.

The main strength of our study was the use of valid and reliable measures of NP and LBP. Specifically, we used a body diagram to identify the region for NP and LBP and the NRS to measure pain intensity, which has been found more reliable and efficient than the Visual Analogue Scale [31]. Furthermore, the survey was completed using the same survey instrument across samples and during the same academic period. Therefore, the observed differences in prevalence suggest that the burden of LBP and NP is different across student groups rather than caused by issues related to school terms. The main limitation of our study is the potential for participation bias. Although participants were generally similar to the source population, there were small differences in age, gender, and study year. Specifically, the slightly higher participation rate of females may have led to an overestimation of the prevalence [59].

The results of our study suggest that most students in our samples experienced NP or LBP in any given week. This is important because pain can impact young adults’ physical and mental health [60]. Furthermore, if the pain becomes chronic, it could affect students’ long-term well-being and functioning [21]. Therefore, our study highlights the need for further research to explore the potential causal factors that lead to the onset of LBP or NP in this age group. Furthermore, updating the study may be important following the COVID-19 pandemic [61].

## Conclusion

In conclusion, we found that most post-secondary students in our samples experienced LBP and NP in the past week. Furthermore, the one-week prevalence of NP and LBP was higher among chiropractic students at CMCC and among females. This study should draw attention to school administrators about the burden of NP and LBP in post-secondary students.

## Electronic supplementary material

Below is the link to the electronic supplementary material.

Supplementary Material 1

Supplementary Material 2

## Data Availability

Not available.
