# Supplementary material for: The one-week prevalence of neck pain and low back pain in post-secondary students at two Canadian institutions
Source: Chiropr Man Therap. 2023 Jul 31;31:23. doi: 10.1186/s12998-023-00496-y (PMC10391772; doi:10.1186/s12998-023-00496-y)
Supplement: Supplementary file 2 — Supplementary Material 2 [file 12998_2023_496_MOESM2_ESM.docx]

**Supplementary Materials**

UOIT Mental Health and Wellness Study

Thank you very much for your willingness to participate in this survey. Your answers to the questionnaire will remain confidential. Once completed and submitted, the researchers will not be able to trace your answers back to you. If you agree to participate please check the box which is located at the bottom of the informed consent form.

The purpose of this study is to assess the test feasibility of a conducting a large study to describe the burden and lifestyle factors associated with mental health and wellness in undergraduate university students.

This questionnaire inquires about mental health and wellness and factors that may be related to it. These factors include sleep quality, physical activity, food access, neck and back pain, substance use and

socio-demographic variables.

You must be 18 years or older to participate in this study.

We would like to remind you that if you are concerned about your well-being, or feel that, you may benefit from support and assistance, please contact Student Mental Health Services to set up an appointment. You can contact student services in one of three ways:

- email [studentlifeline@uoit.ca](mailto:studentlifeline@uoit.ca)

- call 905.721.3392

- drop by Student Life suite (U5 Building at North Campus or 2nd floor of 61 Charles St for the downtown campus) for a chat.

There are also community resources available 24/7, they are: Distress Centre Durham at 905-430-2522 and Durham Crisis Line at 905-666-0483

Thank you very much for considering participating in this important study which will help us better understand mental health and wellness in university students.

- Required

**Informed Consent**

Title of Research Study:

UOIT-CMCC Mental Health and Wellness study

Researcher(s):

Dr. Pierre Côté, Dr. Victoria Smye, Dr. Robert Weaver, Dr. Efrosini Papaconstantinou, Dr. Jennifer Laffier, Dr. Ellen Vogel, Dr. Tyler Frederick, and Dr. Cindy Malachowski, Kathy Smith, MHSc, Nayantara Hattangadi , Andrew Reynolds, Michael Short and Nancy Flynn

Faculty of Health Sciences,

University of Ontario Institute of Technology Contact number: (905) 721-8668 Ext 3674 Email: [Kathy.smith@uoit.ca](mailto:Kathy.smith@uoit.ca)

You are invited to participate in a research study at the University of Ontario Institute of Technology in the Faculty of Health Sciences and Faculty of Education. This study (REB File # 17-xxxx) has been reviewed by the University of Ontario Institute of Technology Research Ethics Board and has been approved as of Month day 2017 Please read this form carefully, and feel free to ask any questions you might have. If you have any questions about your rights as a participant in this study, please contact the Ethics and Compliance Officer at 905 721 8668 ext 3693 or [compliance@uoit.ca](mailto:compliance@uoit.ca).

Before agreeing to participate in this study, it is important that you read and understand the following explanation of the proposed study procedures. The following information describes the purpose, procedures, benefits, and risks associated with this study. It also describes your right to refuse to participate or withdraw from the study at any time. In order to decide whether you wish to participate in this research study, you should understand enough about its risks and benefits to be able to make an informed decision. This is known as the informed consent process.

Please read through this document carefully, and ask Kathy Smith or Dr. Pierre Côté to explain anything that you don’t understand before consenting to this study. Make sure all your questions have been answered to your satisfaction before signing this document.

Purpose and Procedure:

The purpose of this study is to enhance our understanding of lifestyle factors that may be associated with mental health issues and help identify students who may be at risk of developing mental health problems. This information is necessary to mitigate the disabling effects of mental health problems.

The mental health and well being in university students is a public health concern in Canada. However, we know very little about the prevalence of depressive symptoms, anxiety and stress among undergraduate university students. Therefore, we need to investigate these issues to better prevent and manage mental health problems in university students.

We would appreciate if you could work with us in gaining knowledge about mental health and wellness in undergraduate students by consenting to participate in this study and completing an online questionnaire. You will complete this questionnaire once during the class time. The questionnaire will take approximately 15 minutes to complete, and all information provided is confidential.

Time commitment:

The questionnaire is a one-time, only, administration and will take approximately 15 minutes to complete. The one-time administration will be in-class first. However if you are not able to participate in class there will be two follow emails sent that will provide a link to the questionnaire to enable you to complete the questionnaire.

Potential Benefits:

There are no direct benefits for participating in the study. However, participating will provide indirect benefits to the student community by reducing stigma and improving awareness of mental health and wellness.

Potential Risk or Discomforts:

There are no known risks associated with participating in the study. However, participants may experience psychological or emotional discomfort. We remind you that UOIT students who needs support or help should feel free to contact UOIT Student Mental Health services at the Student life suite.

We’re here to support you! UOIT offers a range of services for students to support their positive mental health, strengthen their resilience, and help them manage the multiple demands of university life. If you are concerned about your well-being, or feel that, you may benefit from support and assistance, please contact Student Mental Health Services to set up an appointment. You can email ([studentlifeline@uoit.ca](mailto:studentlifeline@uoit.ca)), call 905.721.3392 or drop by Student Life suite (U5 Building at North Campus or 2nd floor of 61 Charles St for the downtown campus).

For more information, and to learn about the services offered please visit the Student Mental Health website: <http://studentlife.uoit.ca/mentalhealth/index.php>

**Informed Consent (Continued)**

Storage of Data:

All data and consent forms will be kept on a secure UOIT network, which the UOIT IT Department has assisted with. The IT department at UOIT will have access to the raw data and will remove and destroy all identifiers (Banner ID, UOIT.net login and date of birth). This data will be stored on a secure Google Drive account. Dr. Pierre Côté, Dr. Efrosini Papaconstantinou, Ms. Kathy Smith, Ms. Nayantara Hattangadi, Mr. Andrew Reynolds, Mr. Michael Short and Ms Nancy Flynn will have access to the de-identified amalgamated data but not to the raw data.

Confidentiality:

You will be using your UOIT.net account to login and answer the questionnaire. Once the data is collected, the UOIT net administration will take the data from the first administration of the questionnaire and assign a Study ID. The Study ID will bear no resemblance to any of your personal identifiers. The Study ID will maintain your anonymity. The UOIT net administration will then send a study data file without identifiers to Dr. Pierre Côté; the IT department will also destroy any original data files with identifiers once the study data file has been sent to Dr. Pierre Côté. Data files will be stored within UOITs Google Drive Suite for Education instance, which is hosted by Google.

Anonymity:

The raw data will be de-identified of any Banner ID or UOIT.net login information and replaced with a Study ID. The de-identified files will be sent to the research team to ensure your anonymity is maintained. Neither Dr. Côté, nor any of the researchers, will have information relating to personal identifiers so the release of these findings will be completely anonymous.

Right to Withdraw:

Your participation in this study is completely voluntary and will not affect your standing within this course. You are free to withdraw at any point in time. If you do not wish to take part in the study, you do not need to complete the consent form and may remain seated in the class. If you wish to withdraw after giving informed consent but before submitting the questionnaire, you may do so by leaving the webpage where the questionnaire is available. This data will not be recorded.

You can also withdraw at anytime before November 15, 2017 by contacting Neil Hopkins or Bevin Moolenschot from the UOIT IT Department by emailing [Ask@uoit.net](mailto:Ask@uoit.net). They will delete your data and you information will be used in the analysis.

Compensation for Participation:

There will be no compensation to participants for involvement with this study.

Debriefing and Dissemination of Results:

The results of this study will be completed by December 2017. If you desire to receive information regarding the results of this study, please contact the researchers at (905) 721-8668 Ext 5922 or by email at [kathy.smith@uoit.ca](mailto:kathy.smith@uoit.ca) or [pierre.cote@uoit.ca](mailto:pierre.cote@uoit.ca). You will also be invited to a debriefing session were the results of the study will be presented to participants.

Participant Concerns and Reporting:

This research project has been approved by the University of Ontario Institute of Technology Research Ethics Board (REB File # 17-xxxxx) as of Month day 2017.

If you have any questions concerning the research study, or experience any discomfort related to the study please contact the researcher(s) at (905) 721-8668 Ext 5922 or by email at [kathy.smith@uoit.ca](mailto:kathy.smith@uoit.ca) or [pierre.cote@uoit.ca](mailto:pierre.cote@uoit.ca).

Any questions regarding your rights as a participant, complaints or adverse events may be addressed to Research Ethics Coordinator at [researchethics@uoit.ca](mailto:researchethics@uoit.ca) or (905) 721 8668 ext 3693.

- 1. **Informed Consent *** *Check all that apply.*

I consent to voluntarily take part in the study with the understanding I may withdraw at any time. I have had an opportunity to ask questions and my questions have been answered. I am aware of all the risks and benefits associated with my participation and have read the entire consent form. I am free to ask questions about the study in the future.

- 1. **Secondary Use of Data**

The information collected for this study may be used for secondary research in the future. This could include secondary data analysis, future research studies etc.

*Check all that apply.*

I agree to allow the data collected in the study to be used for future secondary research

**Ready to begin**

Thank you for participating in the University of Ontario Institute of Technology Student Mental Health and Wellness study. Your answers will remain completely confidential. Once completed and submitted, the researchers will not be able to trace your answers back to you. The questionnaire includes questions regarding lifestyle behaviours (sleep habits, physical activity, alcohol and drug use, food access, socio- demographic variables, neck and back pain) on mental health.

**About yourself**

3. How old are you? *

*Mark only one oval.*

18

19

20

21

22

23

24

25

26

27

28

29

30

31

32

33

34

35

36

37

38

39

40

41

42

43

44

45

46

47

48

49

50

51

52

53

54

55

56

57

58

59

60

4. What gender do you identify with? *

*Mark only one oval.*

Female

Male

Transgender Male/Trans Man/Female-to-Male (FTM)

Transgender Female/Trans Woman/Male-to-Female (MTF)

Genderqueer, neither exclusively male nor female (or Gender Fluid, or Non-Binary Gender)

Choose not to disclose

Other:

5. What is your program of study? *

*Mark only one oval.*

Nursing

Kinesiology

Public Health

Human Health

Medical Laboratory Science

Allied Health Science

Health Science Comprehensive

Fitness and Health Promotion Bridge

Nursing (Registered Practical Nurse Bridge)

Other:

6. What is your year of study? *

*Mark only one oval.*

1st year

2nd year

3rd year

4th year

5+ year

7. Have you been diagnosed with any of the following medical conditions by a healthcare

provider?

Please check all that may apply

*Check all that apply.*

Allergies (including hay fever or nasal allergy)

Arthritis, for example osteoarthritis, rheumatoid arthritis, gout or any other type, excluding

fibromyalgia

Asthma

Attention disorder or learning disability (e.g., attention deficit disorder, attention deficit

hyperactivity disorder, learning disability)

Bowel disorder such as Crohn's Disease, ulcerative colitis, Irritable Bowel Syndrome or bowel

incontinence

Chronic fatigue syndrome

Eating disorder (e.g., anorexia nervosa, bulimia nervosa)

High blood pressure

Intestinal or stomach ulcers

Migraine headaches

Mood disorder such as depression, bipolar disorder, mania or dysthymia

Scoliosis

Sexually transmitted infection(s)

Other:

**About your physical activity in the past 7 days**

Please answer the following questions based on what you do in a typical week. To increase accuracy, you may wish to think about your physical activity and sedentary behaviour for one week prior to answering the questions.

Aerobic Physical Activity

8. In a typical week, how many days do you do moderate-intensity (like brisk walking) to

vigorous-intensity (like running) aerobic physical activity ? *

*Mark only one oval.*

1 day

2 days

3 days

4 days

5 days

6 days

7 days

9. On average for days that you do at least moderate-intensity aerobic physical activity

(as specified above), how many minutes do you do?

Please answer the following questions based on what you do in a typical week. To increase accuracy, you may wish to think about your physical activity and sedentary behaviour for one week prior to answering the questions.

Muscle Strengthening Physical Activity

10. In a typical week, how many times do you do muscle strengthening activities (such as resistance training or very heavy gardening)?

Please answer the following questions based on what you do in a typical week. To increase accuracy, you may wish to think about your physical activity and sedentary behaviour for one week prior to answering the questions.

Perceived Aerobic Fitness

11. In general, would you say that your aerobic fitness (ability to walk/run distances) is: *

*Mark only one oval.*

Excellent

Very Good

Good

Fair

Poor

Please answer the following questions based on what you do in a typical week. To increase accuracy, you may wish to think about your physical activity and sedentary behaviour for one week prior to answering the questions.

Sedentary Behaviour

12. On a typical day, how many hours do you spend in continuous sitting: at work, in meetings, volunteer commitments and commuting (i.e., by motorized transport)? *

*Mark only one oval.*

None

less than 1 hour

1 to less than 2 hours

2 to less than 3 hours

3 to less than 4 hours

4 to less than 5 hours

5 to less than 6 hours

more than 6 hours

13. On a typical day, how many hours do you watch television, use a computer, read, and spend

sitting quietly during your leisure time? *

*Mark only one oval.*

None

less than 1 hour

1 to less than 2 hours

2 to less than 3 hours

3 to less than 4 hours

4 to less than 5 hours

5 to less than 6 hours

more than 6 hours

14. When sitting for prolonged periods (one hour or more), at what interval would you typically take a break to stand and move around for two minutes? *

*Mark only one oval.*

less than 10 minutes

10 to less than 20 minutes

20 to less than 30 minutes

30 to less than 45 minutes

45 to less than 1 hour

1 to less than 1.5 hours

1.5 to less than 2 hours

more than 2 hours

**About your Mental Health**

Please read each statement and place a check beside the option which indicates how much the statement applied to you over the past week. There are no right or wrong answers. Do not spend too much time on any statement.

The rating scale is as follows:

- Never: Did not apply to me at all

- Sometime: Applied to me to some degree, or some of the time

- Often: Applied to me to a considerable degree, or a good part of time

- Almost Always: Applied to me very much, or most of the time

15. Your Mental Health *

Check the one best response (below) regarding your mental health in the past week.

*Mark only one oval per row.*

Never/Sometimes/Often/Almost Always

1. I found it hard to wind down
2. I was aware of dryness of my mouth
3. I couldn't seem to experience any positive feeling at all
4. I experienced breathing difficulty (e.g. excessively rapid breathing, breathlessness in the absence of physical exertion)
5. I found it difficult to work up the initiative to do things
6. I tended to over-react to situations
7. I experienced trembling (e.g. in the hands)
8. I felt that I was using a lot of nervous energy
9. I was worried about situations in which I might panic and make a fool of myself
10. I felt that I had nothing to look forward to
11. I found myself getting agitated
12. I found it difficult to relax
13. I felt down-hearted and blue
14. I was intolerant of anything that kept me from getting on with what I was doing
15. I felt I was close to panic
16. I was unable to become enthusiastic about anything
17. I felt I wasn't worth much as a person
18. I felt that I was rather touchy
19. I was aware of the action of my heart in the absence of physical exertion (e.g. sense of heart rate increase, heart missing a beat)
20. I felt scared without any good reason
21. I felt that life was meaningless

16. Please think about the last 30 days, taking both good and bad days into account. For each

question, please tell me how much of a problem it is for you on a scale from 1 to 5. 1 means

no problem and 5 means extreme problem. *

*Mark only one oval per row.*

1/2/3/4/5/Don't Know

1. How much of a problem do you have with feeling sad, low or depressed?
2. How much of a problem do you have with feeling worried, nervous or anxious?
3. How much of a problem is handling stress, such as controlling the important things in your life?
4. How much of a problem is coping with all the things you have to do?

**About your Sleep**

The following questions relate to your usual sleep habits during the past month only. Your answers should indicate the most accurate reply for the majority of days and nights in the past month. Please answer all questions.

17. During the past month when have you USUALLY gone to bed at night? *

Please indicate the hour (Hrs) and minute (Mins) and ensure you have chosen AM or PM.

*Example: 8:30 AM*

18. During the past month, how long has it USUALLY taken you to fall asleep each night? *

*Mark only one oval.*

15 minutes or less

16 - 30 minutes

31 - 60 minutes

more than 60 minutes

19. During the past month, when have you USUALLY gotten up in the morning? *

Please indicate the hour (Hrs) and minute (Mins) and ensure you have chosen AM or PM.

*Example: 8:30 AM*

20. During the past month, how many HOURS of actual sleep did you get a night? (This may be different than the number of hours you spend in bed.) *

Please indicate the number of hours (Hrs) and minutes (Mins).

*Mark only one oval.*

more than 7 hours sleep/night

6 to 7 hours sleep/night

5 to 6 hours sleep/night

less than 5 hours sleep/night

21. During the past month, how often have you had trouble sleeping because you... *

For each of the remaining questions, check the one best response.

*Mark only one oval per row.*

Not during the past month/Less than once a week/Once or twice a week/Three or more times a week

1. Cannot get to sleep within 30 minutes
2. Wake up in the middle of the night or early morning
3. Have to get up to use the bathroom
4. Cannot breathe comfortably
5. Cough or snore loudly
6. Feel too cold
7. Feel too hot
8. Have bad dreams
9. Have pain
10. Other reason(s), please describe, including how often you have had trouble sleeping of this reason(s):

22. If you have checked Other reason(s) above, please describe:

23. During the past month... *

For each of the remaining questions, check the one best response.

*Mark only one oval per row.*

Not during the past month/Less than once a week/Once or twice a week/Three or more times a week

1. During the past month, how often have you taken medicine (prescribed or "over the counter") to help you sleep?
2. During the past month, how often have you had trouble staying awake while driving, eating meals, or engaging in social activity?
3. During the past month, how much of a problem has it been for you to keep up enthusiasm to get things done?

24. During the past month, how would you rate your sleep quality overall? *

*Mark only one oval.*

1. Very good
2. Fairly good
3. Fairly bad
4. Very bad
5. **About your access to Food**

These next questions (statements) are about the food eaten in your household in the last 12 months, since March of last year and whether you were able to afford the food you need. Select the appropriate option from the choices listed below each statement depending on the number of persons in the household.

25. "The food that (I/we) bought just didn’t last, and (I/we) didn’t have money to get more.” * Was that often, sometimes, or never true for (you/your household) in the last 12 months?

*Mark only one oval.*

1. Often true
2. Sometimes true
3. Never true
4. Don't know/refuse to answer

26. “(I/we) couldn’t afford to eat balanced meals.” *

1. Was that often, sometimes, or never true for (you/your household) in the last 12 months?
2. *Mark only one oval.*
3. Often true
4. Sometimes true
5. Never true
6. Don't Know/refuse to answer

27. In the last 12 months, since last March, did you and/or other persons in your household ever cut the size of your meals or skip meals because there wasn't enough money for food? *

1. *Mark only one oval.*
2. Yes *Skip to question 28.*
3. No *Skip to question 29.*
4. Don't know *Skip to question 29.*

28. How often did this happen—almost every month, some months but not every month, or in only 1 or 2 months?

1. *Mark only one oval.*
2. Yes, almost every month
3. Yes, some months but not every month
4. Yes, only 1 or 2 months
5. No

29. In the last 12 months, did you ever eat less than you felt you should because there wasn't enough money for food? *

1. *Mark only one oval.*
2. Yes
3. No
4. Don't Know

30. In the last 12 months, were you ever hungry but didn't eat because there wasn't enough money for food?

1. *Mark only one oval.*
2. Yes
3. No
4. Don't Know

**About your Neck and Back Pain**


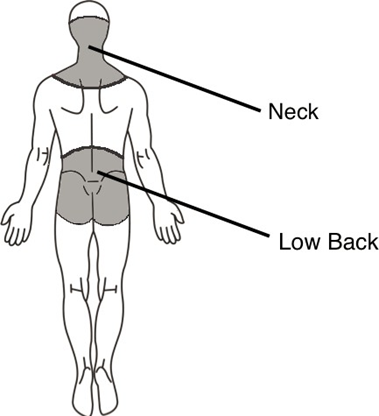


31. In the past 7 days, have you experienced any pain in your NECK? *

*Mark only one oval.*

1. Yes
2. No *After the last question in this section, skip to question 33.*

32. If yes, please indicate the intensity of your average NECK pain over the past 7 days on a scale of 0 (no pain) to 10 (worst pain imaginable).

*Mark only one oval.*

1. 0 1 2 3 4 5 6 7 8 9 10

**About your Low Back Pain**

33. In the past 7 days, have you experienced any pain in your LOW BACK? *

*Mark only one oval.*

Yes

No *After the last question in this section, skip to "About your Alcohol and Drug use."*

34. If yes, please indicate the intensity of your average LOW BACK pain over the past 7 days on a scale of 0 (no pain) to 10 (worst pain imaginable).

*Mark only one oval.*

0 1 2 3 4 5 6 7 8 9 10

**About your Alcohol and Drug use**

This is a brief survey about alcohol, tobacco products and other drugs. Some questions will be asked about your experience of using these substances across your lifetime and in the past three months. These substances can be smoked, swallowed, snorted, inhaled, injected or taken in the form of pills. Some of the substances listed may be prescribed by a doctor (like amphetamines, sedatives, pain medications). Please do not report MEDICATIONS AS PRESCRIBED by your doctor unless used outside the prescription (e.g. increased frequency or higher doses). While we are also interested in knowing about your use of various illicit drugs, please be assured that information on such use will be treated as strictly confidential.

Tobacco use

cigarettes, chewing tobacco, cigars, etc.

35. In your life, have you ever used tobacco products (cigarettes, chewing tobacco, cigars, etc.)? (NON-MEDICAL USE ONLY) *

*Mark only one oval.*

No *Skip to question 41.*

Yes

Tobacco use

cigarettes, chewing tobacco, cigars, etc.

36. In the past 3 months, how often have you used tobacco products (cigarettes, chewing

tobacco, cigars, etc.)? *

*Mark only one oval.*

Never *Skip to question 39.*

Once or Twice

Monthly

Weekly

Daily or Almost Daily

Tobacco use

cigarettes, chewing tobacco, cigars, etc.

37. During the past three months, how often have you had a strong desire or urge to use tobacco products (cigarettes, chewing tobacco, cigars, etc.)?

*Mark only one oval.*

Never

Once or Twice

Monthly

Weekly

Daily or Almost Daily

38. During the past three months, how often has your use of tobacco products (cigarettes,

chewing tobacco, cigars, etc.) led to health, social, legal or financial problems?

*Mark only one oval.*

Never

Once or Twice

Monthly

Weekly

Daily or Almost Daily

Tobacco use

cigarettes, chewing tobacco, cigars, etc.

39. Has a friend or relative or anyone else ever expressed concern about your use of tobacco products (cigarettes, chewing tobacco, cigars, etc.)?

*Mark only one oval.*

No, Never

Yes, in the past 3 months

Yes, but not in the past 3 months

40. Have you ever tried and failed to control, cut down or stop using tobacco products

(cigarettes, chewing tobacco, cigars, etc.)?

*Mark only one oval.*

No, Never

Yes, in the past 3 months

Yes, but not in the past 3 months

Alcohol use

beer, wine, spirits, etc.

41. In your life, have you ever used alcoholic beverages (beer, wine, spirits, etc.)? (NON-MEDICAL USE ONLY) *

*Mark only one oval.*

No *Skip to question 48.*

Yes

Alcohol use

beer, wine, spirits, etc.

42. In the past three months, how often have you used alcoholic beverages (beer, wine, spirits, etc.)? *

*Mark only one oval.*

Never *Skip to question 46.*

Once or Twice

Monthly

Weekly

Daily or Almost Daily

Alcohol use

beer, wine, spirits, etc.

43. During the past three months, how often have you had a strong desire or urge to use

alcoholic beverages (beer, wine, spirits, etc.)?

*Mark only one oval.*

Never

Once or Twice

Monthly

Weekly

Daily or Almost Daily

44. During the past three months, how often has your use of alcoholic beverages (beer, wine, spirits, etc.) led to health, social, legal or financial problems?

*Mark only one oval.*

Never

Once or Twice

Monthly

Weekly

Daily or Almost Daily

45. During the past three months, how often have you failed to do what was normally expected of you because of your use of alcoholic beverages (beer, wine, spirits, etc.)?

*Mark only one oval.*

Never

Once or Twice

Monthly

Weekly

Daily or Almost Daily

Alcohol use

beer, wine, spirits, etc.

46. Has a friend or relative or anyone else ever expressed concern about your use of alcoholic beverages (beer, wine, spirits, etc.)?

*Mark only one oval.*

No, Never

Yes, in the past 3 months

Yes, but not in the past 3 months

47. Have you ever tried and failed to control, cut down or stop using alcoholic beverages (beer, wine, spirits, etc.)?

*Mark only one oval.*

No, Never

Yes, in the past 3 months

Yes, but not in the past 3 months

Cannabis use

marijuana, pot, grass, hash, etc.

48. In your life, have you ever used Cannabis (marijuana, pot, grass, hash, etc.)? (NON-MEDICAL USE ONLY) *

*Mark only one oval.*

No *Skip to question 55.*

Yes

Cannabis use

marijuana, pot, grass, hash, etc.

49. In the past three months, how often have you used Cannabis (marijuana, pot, grass, hash, etc.)? *

*Mark only one oval.*

Never *Skip to question 53.*

Once or Twice

Monthly

Weekly

Daily or Almost Daily

Cannabis use

marijuana, pot, grass, hash, etc

.

50. During the past three months, how often have you had a strong desire or urge to use

Cannabis (marijuana, pot, grass, hash, etc.)?

*Mark only one oval.*

Never

Once or Twice

Monthly

Weekly

Daily or Almost Daily

51. During the past three months, how often has your use of Cannabis (marijuana, pot, grass, hash, etc.) led to health, social, legal or financial problems?

*Mark only one oval.*

Never

Once or Twice

Monthly

Weekly

Daily or Almost Daily

52. During the past three months, how often have you failed to do what was normally expected of you because of your use of Cannabis (marijuana, pot, grass, hash, etc.)?

*Mark only one oval.*

Never

Once or Twice

Monthly

Weekly

Daily or Almost Daily

Cannabis use

marijuana, pot, grass, hash, etc.

53. Has a friend or relative or anyone else ever expressed concern about your use of Cannabis (marijuana, pot, grass, hash, etc.)?

*Mark only one oval.*

No, Never

Yes, in the past 3 months

Yes, but not in the past 3 months

54. Have you ever tried and failed to control, cut down or stop using Cannabis (marijuana, pot, grass, hash, etc.)?

*Mark only one oval.*

No, Never

Yes, in the past 3 months

Yes, but not in the past 3 months

Cocaine use

coke, crack, etc.

55. In your life, have you ever used Cocaine (coke, crack, etc.)? (NON-MEDICAL USE ONLY) *

*Mark only one oval.*

No *Skip to question 62.*

Yes

Cocaine use

coke, crack, etc.

56. In the past three months, how often have you used Cocaine (coke, crack, etc.)? *

*Mark only one oval.*

Never *Skip to question 60.*

Once or Twice

Monthly

Weekly

Daily or Almost Daily

Cocaine use

coke, crack, etc.

57. During the past three months, how often have you had a strong desire or urge to use Cocaine (coke, crack, etc.)?

*Mark only one oval.*

Never

Once or Twice

Monthly

Weekly

Daily or Almost Daily

58. During the past three months, how often has your use of Cocaine (coke, crack, etc.) led to health, social, legal or financial problems?

*Mark only one oval.*

Never

Once or Twice

Monthly

Weekly

Daily or Almost Daily

59. During the past three months, how often have you failed to do what was normally expected of you because of your use of Cocaine (coke, crack, etc.)?

*Mark only one oval.*

Never

Once or Twice

Monthly

Weekly

Daily or Almost Daily

Cocaine use

coke, crack, etc.

60. Has a friend or relative or anyone else ever expressed concern about your use of Cocaine (coke, crack, etc.)?

*Mark only one oval.*

No, Never

Yes, in the past 3 months

Yes, but not in the past 3 months

61. Have you ever tried and failed to control, cut down or stop using Cocaine (coke, crack, etc.)?

*Mark only one oval.*

No, Never

Yes, in the past 3 months

Yes, but not in the past 3 months

Amphetamine type stimulant use

speed, diet pills, ecstasy, Adderall, Dexedrine, bennies, uppers, amps, etc.

62. In your life, have you ever used Amphetamine type stimulants (speed, diet pills, ecstasy, Adderall, Dexedrine, bennies, uppers, amps, etc.)? (NON-MEDICAL USE ONLY) *

*Mark only one oval.*

No *Skip to question 69.*

Yes

Amphetamine type stimulant use

speed, diet pills, ecstasy, Adderall, Dexedrine, bennies, uppers, amps, etc.

63. In the past three months, how often have you used Amphetamine type stimulants (speed, diet pills, ecstasy, Adderall, Dexedrine, bennies, uppers, amps, etc.)? *

*Mark only one oval.*

Never *Skip to question 67.*

Once or Twice

Monthly

Weekly

Daily or Almost Daily

Amphetamine type stimulant use

speed, diet pills, ecstasy, Adderall, Dexedrine, bennies, uppers, amps, etc.

64. During the past three months, how often have you had a strong desire or urge to use

Amphetamine type stimulants (speed, diet pills, ecstasy, Adderall, Dexedrine, bennies,

uppers, amps, etc.)?

*Mark only one oval.*

Never

Once or Twice

Monthly

Weekly

Daily or Almost Daily

65. During the past three months, how often has your use of Amphetamine type stimulants (speed, diet pills, ecstasy, Adderall, Dexedrine, bennies, uppers, amps, etc.) led to health, social, legal or financial problems?

*Mark only one oval.*

Never

Once or Twice

Monthly

Weekly

Daily or Almost Daily

66. During the past three months, how often have you failed to do what was normally expected of you because of your use of Amphetamine type stimulants (speed, diet pills, ecstasy, Adderall, Dexedrine, bennies, uppers, amps, etc.)?

*Mark only one oval.*

Never

Once or Twice

Monthly

Weekly

Daily or Almost Daily

Amphetamine type stimulant use

speed, diet pills, ecstasy, Adderall, Dexedrine, bennies, uppers, amps, etc.

67. Has a friend or relative or anyone else ever expressed concern about your use of

Amphetamine type stimulants (speed, diet pills, ecstasy, Adderall, Dexedrine, bennies,

uppers, amps, etc.)?

*Mark only one oval.*

No, Never

Yes, in the past 3 months

Yes, but not in the past 3 months

68. Have you ever tried and failed to control, cut down or stop using Amphetamine type

stimulants (speed, diet pills, ecstasy, Adderall, Dexedrine, bennies, uppers, amps, etc.)?

*Mark only one oval.*

No, Never

Yes, in the past 3 months

Yes, but not in the past 3 months

Inhalant use

nitrous, glue, gas, paint thinner, etc.

69. In your life, have you ever used Inhalants (nitrous, glue, gas, paint thinner, etc.)? (NONMEDICAL USE ONLY) *

*Mark only one oval.*

No *Skip to question 76.*

Yes

Inhalant use

nitrous, glue, gas, paint thinner, etc.

70. In the past three months, how often have you used Inhalants (nitrous, glue, gas, paint thinner, etc.)? *

*Mark only one oval.*

Never *Skip to question 74.*

Once or Twice

Monthly

Weekly

Daily or Almost Daily

Inhalant use

nitrous, glue, gas, paint thinner, etc.

71. During the past three months, how often have you had a strong desire or urge to use

Inhalants (nitrous, glue, gas, paint thinner, etc.)?

*Mark only one oval.*

Never

Once or Twice

Monthly

Weekly

Daily or Almost Daily

72. During the past three months, how often has your use of Inhalants (nitrous, glue, gas, paint thinner, etc.) led to health, social, legal or financial problems?

*Mark only one oval.*

Never

Once or Twice

Monthly

Weekly

Daily or Almost Daily

73. During the past three months, how often have you failed to do what was normally expected of you because of your use of Inhalants (nitrous, glue, gas, paint thinner, etc.)?

*Mark only one oval.*

Never

Once or Twice

Monthly

Weekly

Daily or Almost Daily

Inhalant use

nitrous, glue, gas, paint thinner, etc.

74. Has a friend or relative or anyone else ever expressed concern about your use of Inhalants (nitrous, glue, gas, paint thinner, etc.)?

*Mark only one oval.*

No, Never

Yes, in the past 3 months

Yes, but not in the past 3 months

75. Have you ever tried and failed to control, cut down or stop using Inhalants (nitrous, glue, gas, paint thinner, etc.)?

*Mark only one oval.*

No, Never

Yes, in the past 3 months

Yes, but not in the past 3 months

Sedative or Sleeping Pill use

Valium, Rohypnol, Ativan, Xanax, Clonazepam, downers, tranks, blue heaven, yellow jackets, etc.

76. In your life, have you ever used Sedatives or Sleeping Pills (Valium, Rohypnol, Ativan, Xanax, Clonazepam, etc.)? (NON-MEDICAL USE ONLY) *

*Mark only one oval.*

No *Skip to question 83.*

Yes

Sedative or Sleeping Pill use

Valium, Rohypnol, Ativan, Xanax, Clonazepam, downers, tranks, blue heaven, yellow jackets, etc.

77. In the past three months, how often have you used Sedatives or Sleeping Pills (Valium, Rohypnol, Ativan, Xanax, Clonazepam, downers, tranks, blue heaven, yellow jackets, etc.)? *

*Mark only one oval.*

Never *Skip to question 81.*

Once or Twice

Monthly

Weekly

Daily or Almost Daily

Sedative or Sleeping Pill use

Valium, Rohypnol, Ativan, Xanax, Clonazepam, downers, tranks, blue heaven, yellow jackets, etc.

78. During the past three months, how often have you had a strong desire or urge to use

Sedatives or Sleeping Pills (Valium, Rohypnol, Ativan, Xanax, Clonazepam, downers, tranks, blue heaven, yellow jackets, etc.)?

*Mark only one oval.*

Never

Once or Twice

Monthly

Weekly

Daily or Almost Daily

79. During the past three months, how often has your use of Sedatives or Sleeping Pills (Valium, Rohypnol, Ativan, Xanax, Clonazepam, downers, tranks, blue heaven, yellow jackets, etc.) led

to health, social, legal or financial problems?

*Mark only one oval.*

Never

Once or Twice

Monthly

Weekly

Daily or Almost Daily

80. During the past three months, how often have you failed to do what was normally expected of you because of your use of Sedatives or Sleeping Pills (Valium, Rohypnol, Ativan, Xanax,

Clonazepam, downers, tranks, blue heaven, yellow jackets, etc.)?

*Mark only one oval.*

Never

Once or Twice

Monthly

Weekly

Daily or Almost Daily

Sedative or Sleeping Pill use

Valium, Rohypnol, Ativan, Xanax, Clonazepam, downers, tranks, blue heaven, yellow jackets, etc.

81. Has a friend or relative or anyone else ever expressed concern about your use of Sedatives or Sleeping Pills (Valium, Rohypnol, Ativan, Xanax, Clonazepam, downers, tranks, blue heaven, yellow jackets, etc.)?

*Mark only one oval.*

No, Never

Yes, in the past 3 months

Yes, but not in the past 3 months

82. Have you ever tried and failed to control, cut down or stop using Sedatives or Sleeping Pills (Valium, Rohypnol, Ativan, Xanax, Clonazepam, downers, tranks, blue heaven, yellow jackets, etc.)?

*Mark only one oval.*

No, Never

Yes, in the past 3 months

Yes, but not in the past 3 months

Hallucinogen use

LSD, acid, mushrooms, PCP, Special K, etc.

83. In your life, have you ever used Hallucinogens (LSD, acid, mushrooms, PCP, Special K, etc.)?

(NON-MEDICAL USE ONLY) *

*Mark only one oval.*

No *Skip to question 90.*

Yes

Hallucinogen use

LSD, acid, mushrooms, PCP, Special K, etc.

84. In the past three months, how often have you used Hallucinogens (LSD, acid, mushrooms, PCP, Special K, etc.)? *

*Mark only one oval.*

Never *Skip to question 88.*

Once or Twice

Monthly

Weekly

Daily or Almost Daily

Hallucinogen use

LSD, acid, mushrooms, PCP, Special K, etc.

85. During the past three months, how often have you had a strong desire or urge to use

Hallucinogens (LSD, acid, mushrooms, PCP, Special K, etc.)?

*Mark only one oval.*

Never

Once or Twice

Monthly

Weekly

Daily or Almost Daily

86. During the past three months, how often has your use of Hallucinogens (LSD, acid,

mushrooms, PCP, Special K, etc.) led to health, social, legal or financial problems?

*Mark only one oval.*

Never

Once or Twice

Monthly

Weekly

Daily or Almost Daily

87. During the past three months, how often have you failed to do what was normally expected of you because of your use of Hallucinogens (LSD, acid, mushrooms, PCP, Special K, etc.)?

*Mark only one oval.*

Never

Once or Twice

Monthly

Weekly

Daily or Almost Daily

Hallucinogen use

LSD, acid, mushrooms, PCP, Special K, etc.

88. Has a friend or relative or anyone else ever expressed concern about your use of

Hallucinogens (LSD, acid, mushrooms, PCP, Special K, etc.)?

*Mark only one oval.*

No, Never

Yes, in the past 3 months

Yes, but not in the past 3 months

89. Have you ever tried and failed to control, cut down or stop using Hallucinogens (LSD, acid, mushrooms, PCP, Special K, etc.)?

*Mark only one oval.*

No, Never

Yes, in the past 3 months

Yes, but not in the past 3 months

Opioid use

heroin, morphine, methadone, codeine, Percocet, Fentanyl, Ts, cody, vike, etc.

90. In your life, have you ever used Opioids (heroin, morphine, methadone, codeine, Percocet, Fentanyl, Ts, cody, vike, etc.)? (NON-MEDICAL USE ONLY) *

*Mark only one oval.*

No *Skip to question 97.*

Yes

Opioid use

heroin, morphine, methadone, codeine, Percocet, Fentanyl, Ts, cody, vike, etc.

91. In the past three months, how often have you used Opioids (heroin, morphine, methadone, codeine, Percocet, Fentanyl, Ts, cody, vike, etc.)? *

*Mark only one oval.*

Never *Skip to question 95.*

Once or Twice

Monthly

Weekly

Daily or Almost Daily

Opioid use

heroin, morphine, methadone, codeine, Percocet, Fentanyl, Ts, cody, vike, etc.

92. During the past three months, how often have you had a strong desire or urge to use Opioids (heroin, morphine, methadone, codeine, Percocet, Fentanyl, Ts, cody, vike, etc.)?

*Mark only one oval.*

Never

Once or Twice

Monthly

Weekly

Daily or Almost Daily

93. During the past three months, how often has your use of Opioids (heroin, morphine, methadone, codeine, Percocet, Fentanyl, Ts, cody, vike, etc.) led to health, social, legal or financial problems?

*Mark only one oval.*

Never

Once or Twice

Monthly

Weekly

Daily or Almost Daily

94. During the past three months, how often have you failed to do what was normally expected of you because of your use of Opioids (heroin, morphine, methadone, codeine, Percocet, Fentanyl, Ts, cody, vike, etc.)?

*Mark only one oval.*

Never

Once or Twice

Monthly

Weekly

Daily or Almost Daily

Opioid use

heroin, morphine, methadone, codeine, Percocet, Fentanyl, Ts, cody, vike, etc.

95. Has a friend or relative or anyone else ever expressed concern about your use of Opioids

(heroin, morphine, methadone, codeine, Percocet, Fentanyl, Ts, cody, vike, etc.)?

*Mark only one oval.*

No, Never

Yes, in the past 3 months

Yes, but not in the past 3 months

96. Have you ever tried and failed to control, cut down or stop using Opioids (heroin, morphine, methadone, codeine, Percocet, Fentanyl, Ts, cody, vike, etc.)?

*Mark only one oval.*

No, Never

Yes, in the past 3 months

Yes, but not in the past 3 months

Injection

97. Have you ever used any drug by injection? (NON-MEDICAL USE ONLY) *

*Mark only one oval.*

No, never

Yes, in the past 3 months

Yes, but not in the past 3 months

**Student Life and Experience**

The following section will ask questions about how you feel about your universities policies and environment, and about your relationships with other people.

98. I find university's learning environment conducive. *

Conducive meaning: favourable, beneficial, advantageous, encouraging etc

*Mark only one oval.*

Never

Sometimes

Always

99. I find academic policies of my university student-friendly. *

*Mark only one oval.*

Never

Sometimes

Always

100. I have no close relationships that make me feel good. *

*Mark only one oval.*

Strongly Disagree

Disagree

Agree

Strongly Agree

101. There is no one I feel comfortable talking about my problems with. *

*Mark only one oval.*

Strongly Disagree

Disagree

Agree

Strongly Agree

**Demographic information**

General information

102. What is your marital status? *

*Mark only one oval.*

Single, never married

Married/Common law

Separated/Divorced

Widowed

103. Number of Dependents *

Dependent is a person who relies on another person for support (especially financial support)

*Mark only one oval.*

0 - None

1

2

3

4

5

6 or more

104. What was your academic average in your last year? *

If you are in 1st year, then report the average for your last year of high school. If you are in 2nd year, then report the average for your 1st year.

*Mark only one oval.*

below 60

between 60 to 65

between 66 to 69

between 70 to 75

between 76 to 79

between 80 to 85

between 86 to 89

between 90 to 95

between 95 to 100

105. What is your annual personal income?

*Mark only one oval.*

$0 - $4,999

$5,000 - $9,999

$10,000 - $19,999

Above $20,000

106. What is your households' annual combined personal income?

*Mark only one oval.*

$0 - $49,999

$50,000 - $59,999

$60,000 - $79,999

Above $80,000

107. How many hours a week do you work for pay? *

During the academic calendar year (i.e. September - April)

*Mark only one oval.*

0

1 - 9 hours

10 - 19 hours

20 - 29 hours

30 - 39 hours

more than 40 hours

108. What is your current household living arrangement? *

During the academic calendar year (i.e. September - April)

*Check all that apply.*

Living with relatives

Living with non-relatives (roommates/housemates)

Living in a student residence

Living alone

Living with a partner

109. On average how long is your commute time to the University? *

*Mark only one oval.*

Less than 15 minutes

15 to 29 minutes

30 to 44 minutes

45 minutes or more

110. Were you born in Canada? *

*Mark only one oval.*

Yes *Skip to question 111.*

No

111. Please check if you are registered at the University as: *

*Mark only one oval.*

An International student

A Domestic student

112. What were the ethnic or cultural origins of your ancestors? *

An ancestor is usually more distant than a grandparent.

*Check all that apply.*

Aboriginal/First Nations/Métis

Black

Caucasian

East Asian

South Asian

South East Asian

Latin American

Middle Eastern

Don’t know

Other:

113. What is your parents marital status? *

*Mark only one oval.*

Single, never married

Separated/Divorced

Married/Common law

Widowed

114. What is the employment status of your primary guardian? *Please check all that apply *

*Check all that apply.*

Full-time employment

Part-time employment

Homemaker

Temporary or seasonal work

Retired

Disability leave

Unemployed

Student

Not applicable

I Don't know

115. What is the employment status of your secondary guardian? *Please check all that apply *

*Check all that apply.*

Full-time employment

Part-time employment

Homemaker

Temporary or seasonal work

Retired

Disability leave

Unemployed

Student

Not applicable

I Don't know
